# 7,8-Dihydroxyflavone Attenuates Inflammatory Response and Insulin Resistance Induced by the Paracrine Interaction between Adipocytes and Macrophages

**DOI:** 10.3390/ijms24043520

**Published:** 2023-02-09

**Authors:** Ye-Eun Shin, Ji Won Choi, Yong Il Park, Hye-Kyeong Kim

**Affiliations:** 1 Department of Food Science & Nutrition, The Catholic University of Korea, Bucheon 14662, Republic of Korea; 2 Department of Biotechnology, Graduate School, The Catholic University of Korea, Bucheon 14662, Republic of Korea

**Keywords:** 7,8-dihydroxyflavone, adipocyte, macrophage, inflammation, insulin resistance

## Abstract

Obesity-induced inflammation and insulin resistance are mediated by macrophage infiltration into adipose tissue. We investigated the effects of 7,8-dihydroxyflavone (7,8-DHF), a flavone found in plants, on the inflammatory response and insulin resistance induced by the interaction between adipocytes and macrophages. Hypertrophied 3T3-L1 adipocytes were cocultured with RAW 264.7 macrophages and treated with 7,8-DHF (3.12, 12.5, and 50 μM). The inflammatory cytokines and free fatty acid (FFA) release were evaluated by assay kits, and signaling pathways were determined by immunoblotting. Coculture of adipocytes and macrophages increased inflammatory mediators, such as nitric oxide (NO), monocyte chemoattractant protein-1 (MCP-1), tumor necrosis factor-alpha (TNF-α), and interleukin-6 (IL-6) and FFA secretion but suppressed the production of anti-inflammatory adiponectin. 7,8-DHF counteracted the coculture-induced changes (*p* < 0.001). 7,8-DHF also inhibited c-Jun N-terminal kinase (JNK) activation and blocked nuclear factor kappa B (NF-κB) nuclear translocation in the coculture system (*p* < 0.01). In addition, adipocytes cocultured with macrophages did not increase glucose uptake and Akt phosphorylation in response to insulin. However, 7,8-DHF treatment recovered the impaired responsiveness to insulin (*p* < 0.01). These findings show that 7,8-DHF alleviates inflammation and adipocyte dysfunction in the coculture of hypertrophied 3T3-L1 adipocytes and RAW 264.7 macrophages, indicating its potential as a therapeutic agent for obesity-induced insulin resistance.

## 1. Introduction

The prevalence of obesity is increasing markedly worldwide [1]. Obesity is a primary health risk factor closely linked to the development of insulin resistance and serious chronic diseases [2]. Insulin resistance, the defective responsiveness of the peripheral tissues to insulin, results in hyperglycemia and leads to type 2 diabetes (T2DM) and its complications, such as cardiovascular disease, retinopathy, and chronic kidney disease [3]. Obesity is characterized by an excess of adipose tissue and chronic low-grade inflammation that is considered to play a causative role in inducing insulin resistance [4,5,6].

Obesity-associated inflammation initiates in adipose tissue [7]. Adipocytes become hypertrophic in obesity, which increases basal lipolysis and the release of inflammatory cytokines, such as interleukin (IL)-6 and monocyte chemoattractant protein-1 (MCP-1) [8]. MCP-1 and free fatty acids (FFA) from hypertrophied adipocytes recruit circulating macrophages [9]. In addition to the increased infiltration of macrophages, adipose tissue macrophages switch to a pro-inflammatory M1 phenotype and secrete various inflammatory cytokines, including tumor necrosis factor-alpha (TNF-α), IL-6, and IL-1β [10,11]. In turn, macrophage-derived inflammatory factors, such as TNF-α, induce lipolysis by interfering with adipocyte insulin signaling [12]. This paracrine interaction between adipocytes and macrophages contributes to an inflammatory change and insulin resistance in obese adipose tissue [13]. Thus, targeting the interaction between adipocytes and macrophages to mitigate inflammation might be a therapeutic strategy in the treatment of obesity-induced insulin resistance.

Currently, insulin and several types of drugs, including sulfonylureas, thiazolidinediones, and biguanides, are used for T2DM treatment. However, these drugs have undesirable side effects, such as hypoglycemia, weight gain, pain, and increased risk of bone fracture [14]. Thus, research efforts are directed toward bioactive constituents from plants as a complementary approach in the management of T2DM [15]. Considering that chronic inflammation is one of the main causes of insulin resistance, natural anti-inflammatory compounds could be a safe and effective intervention. Flavonoids are abundant in fruits and vegetables and have well-recognized anti-inflammatory properties [16].

**Figure 1:**
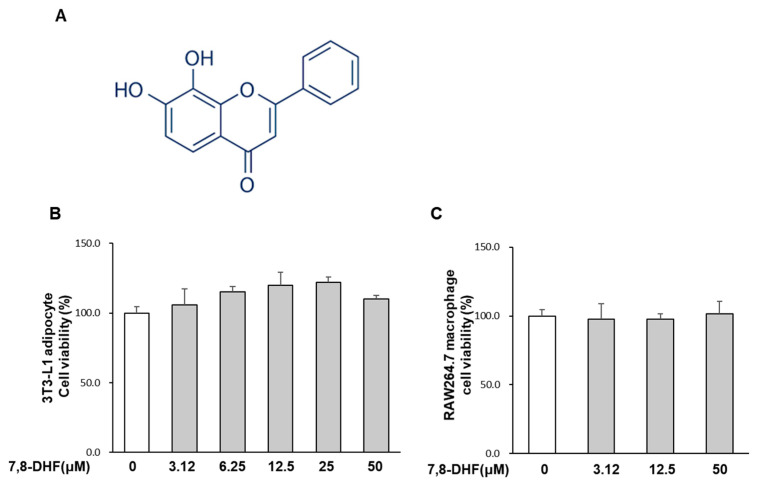
Effect of 7,8-DHF on the viability of 3T3-L1 cells and RAW 264.7 macrophages. (**A**) Structure of 7,8-DHF. (**B**,**C**) Cell viability was assessed using the MTS assay after 24 h exposure to various concentrations of 7,8-DHF (3.12, 12.5, and 50 μM). Results are expressed as a percentage relative to the untreated control cells. Values are mean ± standard deviation (SD) (*n* = 6) and representative of results obtained from three independent experiments. 7,8-DHF, 7,8-dihydroxyflavone.

7,8-dihydroxyflavone (7,8-DHF, Figure 1A) is a flavonoid found in several plants, including the weed *Tridax procumbens* and the tree *Godmania aesculifolia.* 7,8-DHF is slightly soluble in alcohol and acts as a small-molecule agonist (molecular weight, 254.24) of the tropomyosin-related kinase B (TrkB) receptor [17]. Various health benefits of 7,8-DHF have been reported, such as neuroprotective, anti-hypertensive, and anti-obesity effects [18,19,20]. Furthermore, it displays antidiabetic effects, including increasing energy expenditure and reducing ectopic lipid accumulation and fasting blood glucose in obese mice through activating muscular TrkB [20,21], and its anti-inflammatory activity has been demonstrated in macrophages and neural stem cells [22,23]. 7,8-DHF inhibited the secretion of lipopolysaccharide (LPS)-induced inflammatory mediators, such as nitric oxide (NO), prostaglandin E_2_ (PGE_2_), and IL-1β in RAW 264.7 macrophages [22]. However, it remained unclear whether 7,8-DHF can block the paracrine interaction in adipose tissue to attenuate obesity-induced inflammation and insulin resistance.

The aim of this study is to investigate whether 7,8-DHF could have an antidiabetic effect by disrupting the interaction between adipocytes and macrophages in obese adipose tissue. We examined the direct effects of 7,8-DHF on inflammatory mediators, FFA release, and insulin-stimulated glucose uptake in a coculture model of hypertrophied 3T3-L1 adipocytes and RAW 264.7 macrophages.

## 2. Results

### 2.1. Effect of 7,8-DHF on Cell Viability

As shown in Figure 1B,C, 7,8-DHF did not inhibit the viability of hypertrophied mature adipocytes and RAW 264.7 macrophages up to 50 μM. Thus, cells were treated with the non-cytotoxic range of 7,8-DHF in all subsequent experiments.

### 2.2. 7,8-DHF Alleviates Inflammatory Responses in the Coculture of Adipocytes and Macrophages

**Figure 2:**
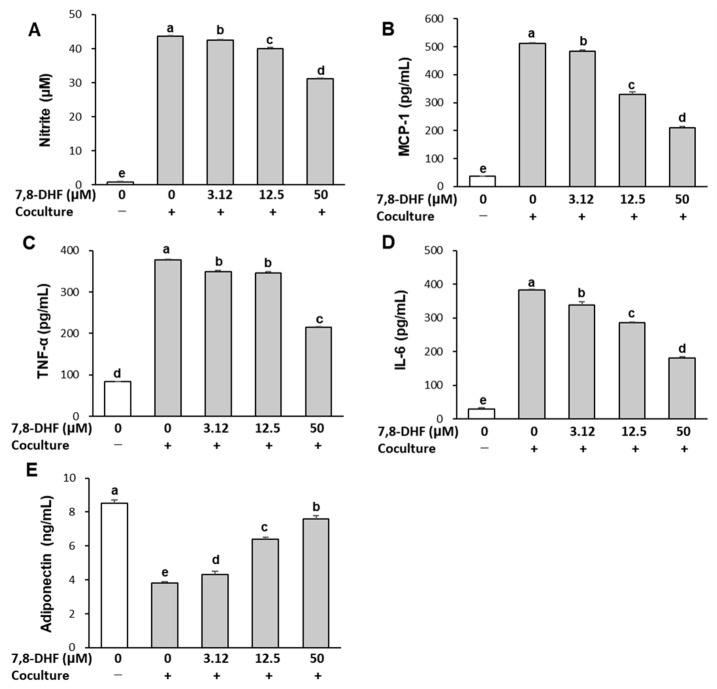
Effect of 7,8-DHF on inflammatory changes in coculture of adipocytes and macrophages. Hypertrophied 3T3-L1 adipocytes were cocultured with RAW 264.7 macrophages for 24 h and then treated with 7,8-DHF for 24 h in the contact system. The concentrations of NO (**A**), MCP-1 (**B**), TNF-α (**C**), IL-6 (**D**), and adiponectin (**E**) were measured in the coculture medium as described in Materials and Methods. Values are means ± SD (*n* = 3) and representative of results obtained from three independent experiments. Means without the same letters are significantly different by ANOVA, followed by Duncan’s test (*p* < 0.001). +: adipocytes were cocultured with macrophages, −: adipocytes and macrophages were separately cultured and mixed before the assay. 7,8-DHF, 7,8-dihydroxyflavone; NO, nitric oxide, MCP-1, monocyte chemoattractant protein-1; TNF-α, tumor necrosis factor-alpha; IL-6, interleukin-6.

The effect of 7,8-DHF on inflammatory responses is shown in Figure 2. The secretion of NO and pro-inflammatory cytokines, including MCP-1, TNF-α, and IL-6, was very low in separately cultured adipocytes and macrophages. Coculture of hypertrophied 3T3-L1 adipocytes and RAW 264.7 macrophages led to a remarkable increase in these levels. However, the coculture-induced increase in NO, MCP-1, TNF-α, and IL-6 was inhibited by 7,8-DHF in a dose-dependent manner (*p* < 0.001). By contrast, adiponectin production was highest (8.53 ± 0.19 ng/mL, 100%) in the separately cultured control and decreased by 56% (3.78 ± 0.06 ng/mL) in the coculture of adipocytes and macrophages. Treatment with 7,8-DHF significantly increased adiponectin secretion by 15% (4.34 ± 0.21 ng/mL), 68% (6.37 ± 0.09 ng/mL), and 100% (7.58 ± 0.18 ng/mL) at 3.12, 12.5, and 50 μM, respectively, compared with cocultured cells (*p* < 0.001). These results demonstrate an anti-inflammatory effect of 7,8-DHF in the coculture-induced inflammatory response.

### 2.3. 7,8-DHF Reduces Lipolysis in Adipocytes Cocultured with Macrophages

**Figure 3:**
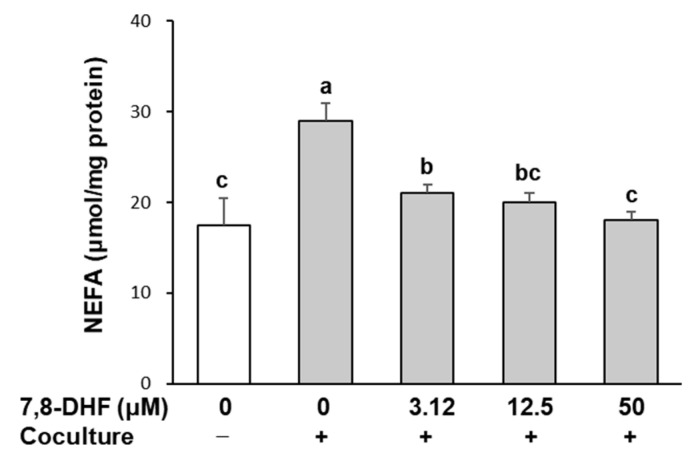
Effect of 7,8-DHF on free fatty acid release in coculture of adipocytes and macrophages. Hypertrophied 3T3-L1 adipocytes were cocultured with RAW 264.7 macrophages for 24 h and then treated with 7,8-DHF for 24 h in the contact system. Concentration of NEFA in the coculture medium was measured by a NEFA kit. Values are mean ± SD of three independent experiments. Means without the same letters are significantly different by ANOVA, followed by Duncan’s test (*p* < 0.001). +: adipocytes were cocultured with macrophages, −: adipocytes and macrophages were separately cultured and mixed before the assay. 7,8-DHF, 7,8-dihydroxyflavone; NEFA, non-esterified fatty acid.

Pro-inflammatory cytokines implicated in obesity-associated adipose tissue inflammation stimulate lipolysis in adipocytes, and chronic FFA release due to stimulated lipolysis in adipocytes is closely associated with insulin resistance [24]. To determine whether 7,8-DHF influences lipolysis, the secreted levels of non-esterified fatty acids (NEFA) into culture media were measured. As shown in Figure 3, NEFA release was elevated by 50% (27 ± 2 μmol/mg) in a coculture of hypertrophied 3T3-L1 adipocytes with RAW 264.7 macrophages compared with the control culture (18 ± 3 μmol/mg). However, this increase was suppressed in response to the treatment with 7,8-DHF (*p* < 0.001). NEFA secretion recovered to the level of the control culture upon treatment with 50 μM 7,8-DHF.

### 2.4. 7,8-DHF Attenuates Nuclear Factor Kappa B (NF-κB) Signaling and c-Jun N-Terminal Kinase (JNK) Activation

**Figure 4:**
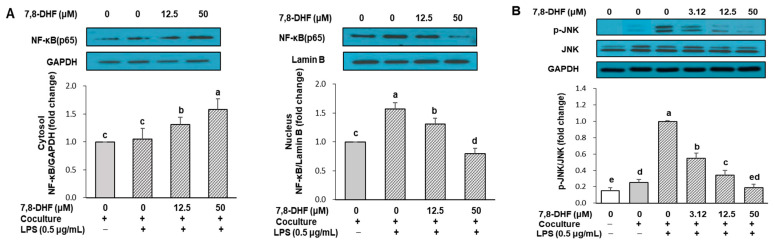
Effect of 7,8-DHF on NF-κB signaling (**A**) and JNK activation (**B**) in coculture of adipocytes and macrophages. Hypertrophied 3T3-L1 adipocytes were cocultured with RAW 264.7 macrophages for 24 h. Cells were treated with 7,8-DHF for 24 h and then stimulated with LPS (0.5 μg/mL) for 30 min. Cytosolic (**left panel**, **A**) and nuclear levels (**right panel**, **A**) of NF-κB p65 subunit were measured to estimate the translocation of NF-κB for activation. Expression of JNK and phosphorylated JNK were measured from total cell lysates by Western blotting. Values are mean ± SD of three independent experiments. Means without the same letters are significantly different by ANOVA, followed by Duncan’s test (*p* < 0.01). Coculture +: adipocytes were cocultured with macrophages, coculture −: adipocytes and macrophages were separately cultured and mixed before the assay. Cells were stimulated with LPS (0.5 μg/mL LPS +) or not (0.5 μg/mL LPS −). 7,8-DHF, 7,8-dihydroxyflavone; NF-κB, nuclear factor kappa B; JNK, c-Jun N-terminal kinase; LPS, lipopolysaccharide; GAPDH, glyceraldehyde-3-phosphate dehydrogenase.

To clarify the mechanisms underlying the anti-inflammatory action of 7,8-DHF in cocultured cells, the NF-κB signaling pathway and JNK phosphorylation were examined. NF-κB is a critical transcription factor that increases the expression of genes encoding pro-inflammatory mediators, such as inducible NO synthase (iNOS), TNF-α, IL-6, and MCP-1 [25]. The primary mechanism in NF-κB activation is that the complex of cytoplasmic inhibitor κB proteins (IκB)—NF-κB is degraded by IκB kinase (IKK) to release NF-κB subunits, leading to NF-κB nuclear translocation and subsequent binding of NF-κB to promoter regions of target genes [25]. We measured the NF-κB p65 subunit in cytosolic and nuclear fractions to estimate the NF-κB translocated from the cytosol to the nucleus. As shown in Figure 4A, LPS treatment to induce IκBα degradation increased the nuclear content of p65 by 1.6-fold. However, when the coculture was treated with 7,8-DHF at concentrations with obvious anti-inflammatory effects (12.5 and 50 μM in the absence of LPS, Figure 2), the nuclear translocation of the NF-κB p65 protein decreased by 16% and 50%, respectively, compared with the LPS-treated coculture control (*p* < 0.01). The effect of 7,8-DHF on the phosphorylation of JNK is shown in Figure 4B. Coculture of hypertrophied adipocytes with macrophages slightly increased the phosphorylation of JNK, which was further stimulated by LPS treatment. However, 7,8-DHF treatment suppressed the LPS-induced phosphorylation of JNK in cocultured cells in a dose-dependent manner (*p* < 0.001). The phosphorylation of JNK recovered to the level of the control culture upon treatment with 50 μM 7,8-DHF.

### 2.5. 7,8-DHF Improves Glucose Uptake in Insulin-Resistant Adipocytes

**Figure 5:**
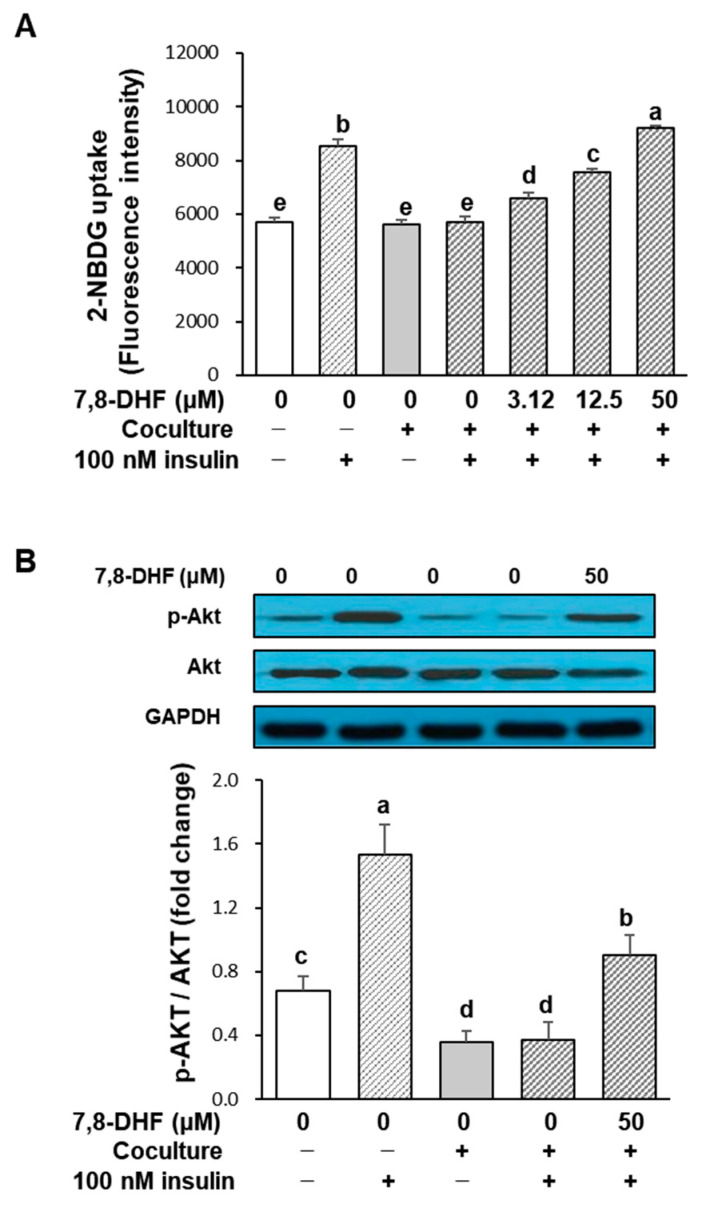
Effect of 7,8-DHF on insulin-induced glucose uptake (**A**) and Akt activation (**B**) in the coculture of adipocytes and macrophages. Hypertrophied 3T3-L1 adipocytes were cocultured with RAW 264.7 macrophages for 24 h and then treated with 7,8-DHF for 6 h. Insulin (100 nM) was added two hours before the end of incubation. For assaying glucose uptake, cells were incubated with a glucose derivative, 2-NBDG, and fluorescence retained in the cell was measured. The protein levels of Akt and phosphorylated Akt were analyzed by Western blotting. Values are mean ± SD, *n* = 6 for glucose uptake and three independent experiments for Akt activation. Means without the same letters are significantly different by ANOVA, followed by Duncan’s test (*p* < 0.01). Coculture +: adipocytes were cocultured with macrophages, coculture −: adipocytes and macrophages were separately cultured and mixed before the assay. Cells were treated with insulin (100 nM insulin +) or not (100 nM insulin −). 7,8-DHF, 7,8-dihydroxyflavone; 2-NBDG, 2-deoxy-2-[(7-nitro-2,1,3-benzoxadiazol-4-yl) amino]-D-glucose; GAPDH, glyceraldehyde-3-phosphate dehydrogenase.

The effect of 7,8-DHF on glucose uptake in the coculture of adipocytes and macrophages is represented in Figure 5A. The incorporation of 2-deoxy-2-[(7-nitro-2,1,3-benzoxadiazol-4-yl) amino]-D-glucose (2-NBDG), a fluorescent glucose derivative, was increased in hypertrophied 3T3-L1 adipocytes without coculture by insulin (fluorescence intensity increased from 3497 ± 160 to 8515 ± 287), while the uptake was not significantly changed in cocultured cells in response to insulin (fluorescence intensity increased from 5618 ± 157 to 5695 ± 210). This result shows that the coculture of hypertrophied 3T3-L1 adipocytes and RAW 264.7 macrophages led to insulin resistance. However, the coculture-induced insulin resistance was recovered upon 7,8-DHF treatment in a dose-dependent manner (*p* < 0.001). 7,8-DHF concentrations of 3.12, 12.5, and 50 μM increased 2-NBDG uptake by 15% (6572 ± 222), 33% (7572 ± 121), and 62% (9192 ± 94), respectively, compared with the insulin-treated coculture control. The 50 μM concentration of 7,8-DHF was sufficient to restore the coculture to the insulin sensitivity of the control adipocytes. To find a possible explanation for the increase in glucose uptake observed in 7,8-DHF-treated adipocytes, we investigated whether the phosphorylation of Akt, a critical factor linking the glucose transporter type four (GLUT4) to the insulin signaling pathway, was involved. As shown in Figure 5B, insulin markedly enhanced the phosphorylation of Akt in the control culture, whereas it did not affect Akt phosphorylation in cocultured cells. Meanwhile, the treatment with 50 μM 7,8-DHF more than doubled the Akt phosphorylation, although it did not reach the level of the insulin-treated control culture. Thus, the recovery of glucose uptake by 7,8-DHF was at least partly ascribed to the increased Akt phosphorylation.

## 3. Discussion

Previous studies on the antidiabetic activity of 7,8-DHF showed that activation of muscular TrkB by 7,8-DHF enhanced energy expenditure, reduced body weight gain, decreased adiposity, and improved insulin sensitivity in vivo [20,21]. Meanwhile, it is well known that adipocyte–macrophage crosstalk in obese adipose tissue plays a causative role in insulin resistance and metabolic dysfunction in obesity [13]. In this study, we examined the effects of 7,8-DHF on inflammatory responses, lipolysis, and insulin-mediated glucose uptake in hypertrophied adipocytes cocultured with macrophages to investigate whether 7,8-DHF can block the paracrine interaction in obese adipose tissue.

Enhanced macrophage infiltration is a feature of obese adipose tissue, and infiltrating macrophages are an important source of inflammation [26]. The coculture system of hypertrophied 3T3-L1 adipocytes and RAW 264.7 macrophages is a useful model of adipose tissue inflammation in which pro-inflammatory cytokines play a causative role [27]. Several flavonoids (luteolin, daidzein, anthocyanins) have been shown to inhibit the production of inflammatory mediators in a coculture system [28,29,30], but the effect of 7,8-DHF remained unexplored. This study found that 7,8-DHF treatment suppressed the coculture-induced upregulation of inflammatory mediators, such as NO, TNFα, IL-6, and MCP-1. MCP-1 stimulates macrophage infiltration into adipose tissue, consequently increasing the expression of cytokines and exacerbating inflammation [9]. Macrophage-derived TNF-α increases the production of pro-inflammatory cytokines and stimulates lipolysis in adipocytes [31]. Hypertrophic adipocytes in obesity show enhanced basal lipolysis, resulting in increased release of FFA [24]. In the present study, NEFA release from adipocytes was further increased when cocultured with macrophages. This phenomenon can be attributed to the enhanced lipolysis by macrophage-derived TNF-α. Adipocyte-derived FFA is another important mediator that activates the production of pro-inflammatory cytokines, in particular MCP-1, which induces macrophage infiltration [32]. Therefore, the reduction in pro-inflammatory cytokines and NEFA release by 7,8-DHF indicates that 7,8-DHF can directly suppress the inflammatory response in obese adipose tissue by disrupting the paracrine loop between adipocytes and macrophages.

Conversely, adiponectin release was decreased in the coculture, and 7,8-DHF treatment restored the coculture-induced suppression of adiponectin. Adiponectin is known to suppress the production of inflammatory cytokines by interfering with the function of macrophages [33]. In addition to its anti-inflammatory action, adiponectin relieves hyperglycemia and improves insulin sensitivity in humans and rodents with obesity by acting on skeletal muscle and the liver [34]. On the contrary, pro-inflammatory cytokines from adipose tissue, such as TNF-α, IL-6, and MCP-1, are known to be associated with insulin resistance [35]. Thus, our results on the inflammatory responses in the coculture of adipocytes and macrophages suggest that 7,8-DHF could improve insulin sensitivity in obese adipose tissue.

The development of obesity-induced inflammation and insulin resistance has been linked to the activation of the JNK and NF-κB pathways [7,36]. Once activated, these signaling pathways induce the production of pro-inflammatory cytokines, which contribute to the inflammatory response. In our study, 7,8-DHF showed a suppressive effect on JNK phosphorylation stimulated by coculture and LPS treatment. LPS also stimulates nuclear translocation of the NF-κB p65 subunit through the degradation of cytoplasmic IκBα, which is essential for NF-κB activation. 7,8-DHF inhibited LPS-induced nuclear translocation of the NF-κB p65 subunit. These results suggest that inhibition of the JNK and NF-κB signaling pathways is a major mechanism for the suppression of inflammatory mediators by 7,8-DHF.

Insulin resistance occurs as a consequence of insulin signaling pathway inhibition, resulting in hyperglycemia due to impaired insulin-mediated glucose uptake and enhanced gluconeogenesis. The binding of insulin to its receptor (IR) on insulin-responsive plasma membranes triggers IR autophosphorylation, which initiates the phosphorylation of tyrosine residues on insulin receptor substrates (IRS), leading to the stimulation of signaling cascades, which promotes the PI3K/Akt signaling pathway [37]. The phosphorylation of Akt is associated with the expression and translocation of GLUT4, a critical mediator of insulin-stimulated glucose uptake in adipocytes [38]. To evaluate the therapeutic efficacy of 7,8-DHF in T2DM by increasing insulin sensitivity, we investigated the effects of 7,8-DHF on 2-NBDG uptake in a coculture model. Contrary to adipocytes cultured separately, adipocytes cocultured with macrophages failed to enhance glucose uptake in response to insulin, which indicates that the coculture induced insulin resistance. We found that 7,8-DHF restored the impaired glucose uptake in the coculture of adipocytes and macrophages, accompanied by increased phosphorylation of Akt. The JNK and NF-κB signaling pathways are involved in insulin resistance as well as the inflammatory response in hypertrophied adipocytes [7]. Activation of both pathways results in the increased production of inflammatory cytokines, such as TNF-α, IL-6, and MCP-1, leading to serine phosphorylation of IRS-1. In addition, JNK signaling can directly inhibit the insulin signaling pathway through phosphorylation of IRS-1 on serine residues. Considering the suppressive effect of 7,8-DHF on the JNK and NF-κB signaling pathways, the recovery of insulin-induced glucose uptake and Akt phosphorylation can be ascribed to the action in the insulin signaling pathway through the reduced production of inflammatory cytokines. Consistent with our result, 7,8-DHF treatment was more effective in reducing blood glucose than water treatment control upon insulin injection in high-fat-induced obese mice [20].

Inferences from this study are limited because we did not examine the in vivo efficacy of 7,8-DHF. Given that animal studies have so far focused on the modulation of blood glucose control, insulin level, and weight reduction after 7,8-DHF treatment, future studies on the alteration of the inflammatory response in adipose tissue of animals with obesity and T2DM are needed to decipher the mechanism by which 7,8-DHF improves insulin sensitivity. Nevertheless, this is the first study to report the direct effect of 7,8-DHF on the inflammatory response and adipocyte metabolic function during the interaction of adipocytes and macrophages.

## 4. Materials and Methods

### 4.1. Materials

7,8-DHF was purchased from Sigma-Aldrich (St. Louis, MO, USA) and dissolved in dimethyl sulfoxide (DMSO, Sigma-Aldrich). Dulbecco’s modified Eagle medium (DMEM), fetal bovine serum (FBS), bovine calf serum (BCS), penicillin-streptomycin, and 2-NBDG were purchased from Invitrogen (Carlsbad, CA, USA). The 3-(4,5-dimethylthiazol-2-yl)-5-(3-carboxy-methoxyphenyl)-2-(4-sulfophenyl)-2H-tetrazolium, Inner Salt (MTS) assay kit was obtained from Promega (Madison, WI, USA). The enzyme-linked immunosorbent assay (ELISA) kits for TNF-α, IL-6, MCP-1, and adiponectin were obtained from R&D Systems (Minneapolis, MN, USA). Nuclear and cytoplasmic extraction reagents and the assay kit for NEFA were provided by Thermo Fisher Scientific (Rockford, IL, USA) and FUJIFILM Wako Pure Chemical Corporation (Chuo-Ku, Osaka, Japan), respectively. Antibodies specific for phospho-c-Jun N-terminal kinases (p-JNK), total-JNK (t-JNK), phospho-Akt (Ser473), Akt, and NF-κB were purchased from Cell Signaling Technology (Danvers, MA, USA). The antibody for lamin B was purchased from Santa Cruz Biotechnology (Santa Cruz, CA, USA), and antibodies for α-tubulin and glyceraldehyde 3-phosphate dehydrogenase (GAPDH) were from Ab Frontier (Seoul, Korea). All other chemicals were obtained from Sigma-Aldrich.

### 4.2. Cell Culture

3T3-L1 mouse embryo fibroblasts and RAW 264.7 macrophage cells were purchased from the American Type Culture Collection (ATCC, Manassas, VA, USA). 3T3-L1 cells were cultured as described previously [30]. Briefly, preadipocytes were grown in DMEM containing 10% BCS until 2 days after confluence and differentiated with induction medium containing 10% FBS, 0.5 µM isobutylmethylxanthine (IBMX), 1 µM dexamethasone, and 167 nM insulin (day 0, D0). After 2 days, cells were maintained in DMEM containing 10% FBS and 167 nM insulin for another 2 days and then cultured in 10% FBS/DMEM. Cells displayed the mature adipocyte phenotype with lipid droplets after approximately day 6 (D6) and continued lipid accumulation thereafter. Adipocytes between day 14 (D14) and day 20 (D20) were used as hypertrophied 3T3-L1 adipocytes. 3T3-L1 adipocytes and RAW 264.7 macrophages were cocultured by a contact system. RAW 264.7 macrophages (5×10^5^ cells/mL) were plated onto serum-starved and hypertrophied 3T3-L1 cells and then incubated in serum-free DMEM for 24 h. As a control culture, 3T3-L1 cells and RAW 264.7 macrophages were cultured separately under the same conditions and mixed 1 h before assays. Cocultured cells were treated with the indicated concentrations of 7,8-DHF or 0.1% DMSO as a control.

### 4.3. Cell Viability Assay (Determination of 7,8-DHF Concentrations for Bioassays)

The MTS assay was used to determine non-toxic concentrations of 7,8-DHF. The concentrations of 7,8-DHF (3.12, 12.5, and 50 μM) were based on previous studies in preadipocytes and macrophages [22,39]. 3T3-L1 cells were seeded in a 96-well plate, cultured to hypertrophied mature adipocyte (D14), and then treated with media containing 7,8-DHF. RAW 264.7 macrophages were seeded in a 96-well plate at a density of 2.5×10^5^ cells/well, and 7,8-DHF was added after 24 h of culture. After incubation for 24 h, the viability of both cells was measured by MTS assay according to the manufacturer’s instructions. The absorbance was measured at 490 nm using a microplate reader (ELx808, Biotek, Winooski, VT, USA) to quantify the formazan concentration, which is proportional to the number of living cells.

### 4.4. Measurement of NO and Cytokine Production

Hypertrophied 3T3-L1 cells and RAW 264.7 macrophages were cocultured for 24 h and then treated with 7,8-DHF (3.12, 12.5, and 50 μM). After 24 h of incubation, the cell culture supernatants were collected and used for assays. The accumulation of nitrite, a stable end product of NO, was quantified by the Griess method. In brief, 100 μL of supernatant was mixed with the same volume of Griess reagent (1% sulfanilamide in 5% phosphoric acid, 0.1% *N*-(1-naphthyl) ethylenediamine in H_2_O). After incubating at room temperature for 10 min, the absorbance at 540 nm was measured using a microplate reader (ELx808, Biotek). The nitrite concentrations were calculated from a standard sodium nitrite curve. The MCP-1, TNF-α, IL-6, and adiponectin concentrations in the culture supernatants were measured using ELISA kits according to the manufacturer’s instructions.

### 4.5. Lipolysis Assay

Lipolysis was determined by measuring the NEFA released to the culture medium from adipocytes. Hypertrophied 3T3-L1 cells were cocultured with RAW 264.7 macrophages for 24 h. After washing with phosphate-buffered saline (PBS), cells were incubated in Krebs–Ringer bicarbonate buffer (119 mM NaCl, 4.8 mM KCl, 1.28 mM CaCl_2_, 1.2 mM KH_2_PO_4_, 1.2 mM 7H_2_O·MgSO_4_, 0.25 mM NaHCO_3_, 5 mM glucose, 4% bovine serum albumin; pH 7.4) containing 7,8-DHF (3.12, 12.5, and 50 μM) for 24 h. The NEFA content in the culture medium was measured by an assay kit, and the protein concentration of the pellets was quantified for calibration.

### 4.6. Glucose Uptake Assay

RAW 264.7 cells were placed onto the hypertrophied 3T3-L1 cells cultured in 96-well plates. After 24 h incubation, cells were preincubated with serum-free DMEM for 2 h and treated with 7,8-DHF (3.12, 12.5, and 50 μM) for 6 h. At 2 h before the end of incubation, insulin (100 nM) was added with 20 µM of the fluorescent glucose derivative, 2-NBDG. After incubation, the treated cells were washed with cold PBS to remove free 2-NBDG, and the fluorescence retained in the cell monolayers was measured at an excitation wavelength of 465 nm and an emission wavelength of 540 nm using a fluorescence microplate reader.

### 4.7. Western Blot Analysis

After hypertrophied 3T3-L1 cells and RAW 264.7 macrophages were cocultured for 24 h, cells were treated with the indicated concentrations of 7,8-DHF. For the assessment of JNK and NF-κB activation, LPS (0.1 μg/mL) was added 30 min before the end of treatment. Cytoplasmic and nuclear fractions were separated using extraction reagents to determine the translocation of NF-κB. Briefly, the treated cells were washed with cold PBS and centrifuged at 500× *g* for 3 min. The cell pellet was fully suspended in cytoplasmic extraction reagent I using a vortex mixer, then kept on ice for 10 min, followed by the addition of cytoplasmic extraction reagent II with mixing using a vortex mixer for 5 s. After incubating on ice for 1 min, the suspension was centrifuged at 16,000× *g* for 5 min. The supernatant fraction (cytoplasmic extract) was transferred to a pre-chilled tube, and the insoluble pellet fraction was resuspended in nuclear extraction reagent, kept on ice for 10 min, and then centrifuged at 16,000× *g* for 10 min to obtain the nuclear extract. For the measurement of Akt activation, insulin (100 nM) was added 2 h before the end of incubation. The cells were collected and lysed in a cold RIPA buffer (20 mM Tris-HCl, 150 mM NaCl, 1 mM Na_2_EDTA, 1 mM EGTA, 1% NP-40, 1% sodium deoxycholate, 2.5 mM sodium pyrophosphate, 1 mM β-glycerophosphate, 1 mM Na_3_VO_4_, and 1 μg/mL leupeptin; pH 7.4) and kept on ice for 15 min. After centrifugation at 15,000× *g* at 4 °C for 30 min, the proteins were separated by 12% SDS-PAGE and transferred to nitrocellulose membranes. The membranes were blocked with 5% skim milk in PBS/0.1% Tween 20 for 1 h and then incubated overnight with the indicated primary antibodies at 4 °C. After washing with PBS, the membrane was incubated with a secondary horseradish peroxidase (HRP)-conjugated antibody for 2 h at room temperature. Immunoblots were visualized by an enhanced chemiluminescence (ECL) system (Ab Frontier) and quantified using a FluorChem densitometer and the ImageJ program (National Institutes of Health [NIH], Bethesda, MD, USA).

### 4.8. Statistical Analysis

Data were analyzed by one-way ANOVA, followed by Duncan’s multiple range test using SAS software (version 9.4; SAS Institute, Inc., Cary, NC, USA), with a significance level of *p* < 0.05. The results were presented as means ± standard deviation (SD).

## 5. Conclusions

We demonstrated that 7,8-DHF suppressed pro-inflammatory changes and lipolysis but restored impairment of insulin-stimulated glucose uptake in hypertrophied adipocytes cocultured with macrophages. The effects of 7,8-DHF were mediated by the inhibition of the NF-κB signaling pathway and JNK activation. These results suggest the therapeutic potential of phytochemical 7,8-DHF in alleviating obesity-induced insulin resistance by disrupting the interaction between adipocytes and macrophages that promotes inflammatory changes and adipocyte dysfunction.

## Data Availability

Data are contained within the article.
